# Repeated Impact Damage Behavior and Damage Tolerance of Bio-Inspired Helical-Structured Glass Fiber Resin Matrix Composites

**DOI:** 10.3390/polym17131720

**Published:** 2025-06-20

**Authors:** Liang He, Zhaoyue Yao, Lanlan Jiang, Zaoyang Guo, Qihui Lyu

**Affiliations:** 1 School of Science, Harbin Institute of Technology, Shenzhen 518055, China; hel@cclmotors.com (L.H.); yaozhaoyue@hit.edu.cn (Z.Y.); jiang_llan@163.com (L.J.); 2 Research Institute of Interdisciplinary Science, School of Materials Science and Engineering, Dongguan University of Technology, Dongguan 523808, China; 3 School of Aerospace, Xi’an Jiaotong University, Xi’an 710049, China

**Keywords:** resin matrix composites, impact behavior, finite element analysis, damage tolerance

## Abstract

This study proposes a bionic helical configuration design concept, focusing on glass-fiber-reinforced polymer matrix composites. Through a combination of experimental and numerical simulation methods, it systematically investigates the resistance to multiple impacts and damage tolerance. The research designs and fabricates two types of bionic laminates: a cross-helical and a symmetric-helical structures. By conducting repeated impact experiments at 5 J of energy for 1, 5, 10, and 15 impact times and employing advanced characterization techniques, such as ultrasonic C-scan and X-ray CT, the study reveals the mechanisms of interlaminar damage propagation and failure characteristics. Based on experimental findings, a finite element model encompassing the entire impact process and post-impact compression behavior is established. Utilizing this model, three optimized novel bionic configurations are further developed, providing new insights and theoretical support for the structural design of high-performance impact-resistant polymer matrix composites.

## 1. Introduction

Glass-fiber-reinforced composites (GFRCs) have been extensively applied in high-tech industries such as aerospace, transportation, and marine engineering due to their superior mechanical properties. These materials demonstrate exceptional impact resistance and excellent damage tolerance under impact-loading conditions [1,2]. Given these characteristics, a systematic investigation into the damage evolution mechanisms and failure modes of GFRCs under impact loading not only provides critical insights for theoretical advancements but also offers significant guidance for practical engineering applications.

Donough et al. [3] characterized the damage behavior of glass-fiber-reinforced composite laminates under in-plane and oblique impacts, providing an in-depth revelation of the initiation and propagation mechanisms of damage in this material under impact loads at different angles. Cesim et al. [4] systematically investigated the low-velocity impact damage behavior of glass fiber-reinforced composites under varying impact energies. They conducted a detailed analysis of the evolution process from initial damage to final perforation, determined the penetration threshold, penetration range, and perforation threshold, and elucidated the correlation between these key impact parameters and damage progression. Nassir et al. [5] investigated the mechanical response of glass-fiber-reinforced composites under low-velocity impact, revealing that the peak load and absorbed energy increase with a higher impact energy, while the absorbed energy decreases significantly with an increasing impact velocity, showing no significant correlation between the peak load and impact velocity. Amaro et al. [6] examined the behavior of glass-fiber-reinforced composites under single and multiple impacts at the same total energy level, exploring the effects of repeated low-velocity impacts at different energy levels on the performance of glass/epoxy laminates.

Guha et al. [7] investigated piezoelectric-fiber-reinforced composites through micromechanical modeling and numerical techniques, demonstrating that the fiber volume fraction significantly influences composite performance. Their findings highlight the critical role of fiber architecture optimization in enhancing the mechanical properties of laminates. In recent years, the rise of bio-inspired structural design has opened new avenues for enhancing the impact resistance and damage tolerance of composite materials. Liu et al. [8] demonstrated that incorporating a bio-inspired helical ply design can further improve the impact performance of laminated composites. Chen et al. [9], inspired by the helicoidal structure of the mantis shrimp’s dactyl club periodic region, investigated the differences in impact resistance of laminates with varying ply-angle configurations under different impact velocities. Inspired by the fish scale design, Shreya et al. [10] developed two configurations of fiber-reinforced composite laminates: single-helix and double-helix, and they revealed the differences in the mechanical properties and energy absorption mechanisms between the configurations. Zhao et al. [11] drew inspiration from the mandible structure of trap-jaw ants to design and fabricate a carbon fiber biomimetic laminate with a double-sinusoidal helical structure. Through low-velocity impact tests, computed tomography, and post-impact compression tests, they comprehensively evaluated its impact resistance and damage tolerance.

The bio-inspired helical composite laminate demonstrates significantly enhanced impact resistance compared to conventional cross-ply and unidirectional laminates. Zhang et al. [12] combined the sinusoidal fiber structure of mantis shrimp with a helical fiber arrangement to design and fabricate a novel composite configuration. Compared to traditional laminated composites, this bioinspired composite exhibited a 45% improvement in impact toughness and effectively suppressed the failure caused by stress concentration during impact. Experimental results from Yang et al. [13] demonstrated a ~30% enhancement in the peak load capacity of bio-mimetic spiral composite laminates over traditional configurations under low-velocity impact. Guan et al. [14] showed that Bouligand composites outperform unidirectional counterparts in impact energy absorption, with 40° helical configurations exhibiting 140% higher maximum capacity. Yang et al. [15] revealed that under low-velocity impact, bio-inspired spiral composites exhibited a 49.66% reduction in the damage area and 10.10% higher residual strength compared to unidirectional composites, demonstrating superior damage resistance and structural tolerance.

The above analysis reveals evident limitations in current research on glass fiber composite materials: on one hand, existing studies primarily focus on the single-impact response of traditional laminated layups, with a notable lack of research on the cumulative damage mechanisms under multiple impacts. On the other hand, most current research concentrates on evaluating the impact resistance and damage tolerance of conventional configurations, while neglecting the innovative exploration of bioinspired structural designs. Therefore, there is an urgent need to conduct research on the multiple-impact damage mechanisms of glass fiber composites based on bioinspired structural designs, in order to break through the performance bottlenecks of traditional materials in terms of impact resistance.

This study utilized unidirectional glass fiber prepreg to design and fabricate two types of bionic laminate configurations—cross-spiral and symmetric-spiral—through manual layup and compression molding processes. The dynamic response and residual performance of these two structures under 5 J of impact energy were systematically investigated after 1, 5, 10, and 15 repeated impacts. By employing advanced characterization techniques, such as ultrasonic C-scan and X-ray CT, the mechanisms of interlaminar damage propagation and failure characteristics in different configurations were thoroughly analyzed. Based on experimental research, a finite element model encompassing the entire impact process and post-impact compression behavior was established. Furthermore, this model was extended to evaluate the impact resistance of three bionic laminate configurations, providing important theoretical foundations and technical support for the structural optimization and engineering applications of bionic spiral composites.

## 2. Material and Experimental Procedure

### 2.1. Composite Material and Manufacturing

**Figure 1:**
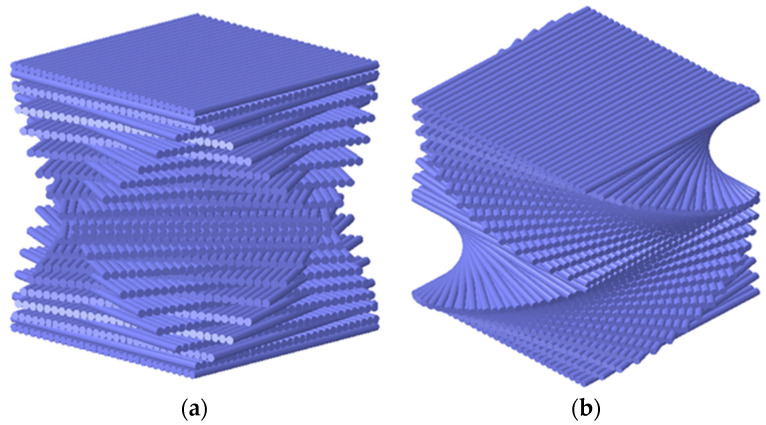
Computational visualization of composite laminate stacking sequences: (**a**) CS (cross-spiral); (**b**) SS (symmetric-spiral).

The impact specimens were fabricated using unidirectional prepreg provided by Zhangjiagang Weinuo Composite Materials Co., Ltd., Suzhou, China, through a manual layup process. The laminates were compression-molded using an XL-3-17-1 hot press (Xuli Electromechanical Equipment Co., Ltd., Dongguan, China) under a staged cure cycle: 90 °C (porosity reduction) → 100 °C (resin permeation) → 120 °C (shape fixation) → 130 °C (full curing), followed by controlled cooling (3–5 min) to minimize residual stresses prior to demolding. Subsequently, high-pressure water jet cutting technology was employed to cut the composite into standard specimens measuring 150 mm× 100 mm× 4.48 mm, in accordance with ASTM D7136 [16]. Two configurations were ultimately produced: bionic cross-helical laminates and bionic symmetric-helical laminates. The layup patterns are illustrated in Figure 1. The details of the two laminates are shown in Table 1. The glass fiber had an areal density of 242 g/m^2^ and a volume fraction of 62%. YPH-69 epoxy resin was employed as the matrix material, characterized by a density of 1.25 g/cm^3^ and representing 38 vol% of the composite. After fabrication, uniaxial tensile, uniaxial compression, and in-plane shear tests were conducted on the glass fiber bionic helical laminates using a WANCE universal tensile testing machine (Shenzhen Wance Testing Co., Ltd., China), following ASTM D3039, D3410, and D7078 standards [17,18,19], respectively. These tests provided material input parameters for the subsequent finite element model, as detailed in Table 2.

### 2.2. Experimental Procedure

This study conducted low-velocity impact tests on two configurations of bionic helical laminates based on the ASTM D7136 standard, using a WANCE drop-weight impact testing machine (Shenzhen Wance Testing Co., Ltd., China). The impact energy was set at 5 J, with 1, 5, 10, and 15 impact times applied, respectively. A smooth hemispherical impactor with a total mass of 5.5 ± 0.25 kg and a diameter of 16 ± 0.1 mm was employed. The impact energy was controlled by adjusting the free-fall height of the impactor, while an anti-rebound system prevented multiple unintended impacts on the specimen. For statistical reliability, a minimum of six parallel specimens were tested per loading condition, yielding at least three valid datasets. During the experiments, built-in sensors in the equipment recorded real-time dynamic parameters, such as the load, displacement, and velocity, providing essential data for analyzing the mechanical response of the bionic helical composite structures under multiple-impact loading.

To accurately analyze internal material defects and precisely measure damage areas, high-resolution ultrasonic C-scan imaging was performed on the laminates using a BSN-C1285 C-scan system (Beijing Sound & Light Technology Co., Ltd., China). The system is equipped with an ultrasonic transducer operating at 5 Hz and features dual-axis (XY) automated scanning with a scanning increment of 0.3 mm. The C-scan images can display varying echo intensities in color or grayscale, effectively visualizing the extent of damage. Finally, ImageJ 1.53t image processing software was employed to quantitatively calculate the damage area from the C-scan results.

The internal damage of bionic helical composite laminates under multiple impacts was effectively characterized using a YXLON FF85 CT system (YXLON International, Heidelberg, Germany). The equipment employed an open-type unipolar X-ray tube coupled with a flat-panel detector to conduct CT scanning of the specimens. The X-ray tube operated at 225 kV with an energy range spanning 20 kV to 16 MV. Each radiograph was acquired with an exposure time of approximately 1.4 s, yielding a total of 3500 radiographic projections. The scan data were subsequently reconstructed and analyzed using VGStudio Max 2024 software.

To investigate the damage tolerance of laminates after multiple impacts, post-impact compression strength tests were conducted using a WANCE microcomputer-controlled electronic universal testing machine (Shenzhen Wance Testing Co., Ltd., China) with a maximum load capacity of 100 kN. The experiments were performed in accordance with the ASTM D7137 standard [21], employing a crosshead displacement rate of 1.25 mm/min. Prior to formal testing, a 150 N preload was applied to the specimen/fixture assembly to ensure full contact between all loading surfaces.

## 3. Numerical Modeling

### 3.1. Composite Damage Model

#### 3.1.1. In-Plane Constitutive Model

When subjected to low-velocity impact, the specimen does not immediately exhibit significant fracture but may develop progressive damage such as microcracks, delamination, and fiber breakage. Therefore, a continuum damage model is employed to describe the gradual propagation of damage during the impact process and to evaluate the final failure mode. The constitutive equation is as follows:

(1) σ=DCε

where σ is the stress tensor, D is the damage tensor, C is the undamaged elastic tensor of the material, and ε is the strain tensor.

Since the fibers in composite laminates typically exhibit brittle failure behavior, this study adopts the maximum strain criterion to assess the initiation of tensile and compressive fiber damage under impact loading. The criterion determines damage onset by defining specific strain thresholds, with the det ailed expressions given as follows:

Fiber tensile mode ($\varepsilon_{11}\geq 0$)

(2) $$F_{\mathrm{ft}}={\left(\frac{\varepsilon_{11}}{X_{T}}E_{11}\right)}^{2}-1\geq 0$$

Fiber compression mode ($\varepsilon_{11}<0$)

(3) $$F_{fc}={\left(\frac{\varepsilon_{11}}{X_{C}}E_{11}\right)}^{2}-1\geq 0$$

Here, $F_{\mathrm{ft}}$ and $F_{\mathrm{fc}}$ represent the initial damage conditions for fiber tension and compression, respectively; $\varepsilon_{11}$ and $E_{11}$ denote the strain along the fiber direction and the elastic modulus of the laminate, respectively; and $X_{T}$ and $X_{C}$ correspond to the tensile and compressive strengths in the fiber direction.

When the strain in the fiber direction of the laminate satisfies Equation (2) or (3), material damage initiates, and the damage variable evolves according to a linear degradation law [22], expressed as follows:

(4) $$d_{\mathrm{ft}(c)}=\frac{\varepsilon_{f,11}^{t(c)}}{\varepsilon_{f,11}^{t(c)}-\varepsilon_{0,11}^{t(c)}}\left(1-\frac{\varepsilon_{0,11}^{t(c)}}{\varepsilon_{11}}\right)$$

Here, $d_{\mathrm{ft}(c)}$ represents the fiber tension/compression damage variable, $\varepsilon_{0,11}^{t(c)}$ denotes the initiation strain for fiber tension/compression damage in the laminate, and $\varepsilon_{f,11}^{t(c)}$ indicates the ultimate failure strain of the laminate in the fiber tension/compression direction.

The damage failure criterion adopted for the matrix under impact loading is as follows [23,24]:

Matrix tensile mode ($\sigma_{\mathrm{NN}}>0$)

(5) $$F_{\mathrm{mt}}={\left(\frac{\sigma_{\mathrm{NN}}}{S_{23}^{is}}\right)}^{2}+{\left(\frac{\tau_{\mathrm{LN}}}{S_{12}^{\mathrm{is}}}\right)}^{2}+{\left(\frac{\tau_{\mathrm{NT}}}{S_{23}^{\mathrm{is}}}\right)}^{2}+\lambda \left(\frac{\sigma_{\mathrm{NN}}}{S_{23}^{\mathrm{is}}}\right){\left(\frac{\tau_{LN}}{S_{12}^{is}}\right)}^{2}+\kappa \left(\frac{\sigma_{\mathrm{NN}}}{S_{23}^{is}}\right)$$

(6) $$\kappa =\frac{{\left(S_{12}^{is}\right)}^{2}-{\left(Y^{T,is}\right)}^{2}}{S_{23}^{is}Y^{T,is}}$$

Matrix compression mode ($\sigma_{\mathrm{NN}}<0$)

(7) $$F_{mc}={\left(\frac{\tau_{LN}}{S_{12}^{is}-\mu_{\mathrm{LN}}\sigma_{\mathrm{NN}}}\right)}^{2}+{\left(\frac{\tau_{NT}}{S_{23}^{\mathrm{is}}-\mu_{\mathrm{NT}}\sigma_{\mathrm{NN}}}\right)}^{2}$$

Here, $F_{mt}$ and $F_{mc}$ represent the initial damage criteria for matrix tension and compression, respectively; $S_{12}^{\mathrm{is}}$ and $S_{23}^{is}$ denote the in situ shear strengths of the matrix in different directions; $Y^{T,is}$ is the in situ transverse tensile strength of the matrix, determined by Equation (7); λ is a constant parameter; and $\sigma_{\mathrm{NN}}$, $\tau_{\mathrm{LN}}$, and $\tau_{\mathrm{NT}}$ correspond to the normal stress and shear stresses on the matrix fracture plane, respectively. A continuum damage evolution model based on equivalent strain is adopted for the composite matrix, and the damage variable is defined as

(8) $$d_{mat}=\frac{\varepsilon_{r}^{f}}{\varepsilon_{r}}\left(\frac{\varepsilon_{r}-\varepsilon_{r}^{0}}{\varepsilon_{r}^{f}-\varepsilon_{r}^{0}}\right)$$

where $\varepsilon_{r}^{f}$ represents the ultimate failure strain of the matrix, and $\varepsilon_{r}^{0}$ denotes the strain at the onset of matrix damage.

#### 3.1.2. Cohesive Constitutive Model

To describe the progressive damage between different plies of the laminate, a bilinear traction-separation constitutive model is introduced, for which the constitutive expression is as follows:

(9) $$t=\left\{\begin{array}{c} t_{n}\\ t_{s}\\ t_{t} \end{array}\right\}=\left[\begin{array}{ccc} K_{nn} & K_{ns} & K_{nt}\\ K_{ns} & K_{ss} & K_{st}\\ K_{nt} & K_{st} & K_{tt} \end{array}\right]\left\{\begin{array}{c} \varepsilon_{n}\\ \varepsilon_{s}\\ \varepsilon_{t} \end{array}\right\}=K\varepsilon$$

Here, ***K*** is the stiffness matrix, ***t*** denotes the traction force, and ε represents the strain. The quadratic nominal stress criterion is adopted to determine the initiation of interlaminar damage, and its constitutive expression is as follows:

(10) $${\left(\frac{\langle t_{n}\rangle }{t_{n}^{0}}\right)}^{2}+{\left(\frac{t_{s}}{t_{s}^{0}}\right)}^{2}+{\left(\frac{t_{t}}{t_{t}^{0}}\right)}^{2}=1$$

where $t_{n}$, $t_{s}$, and $t_{t}$ represent the interlaminar normal traction and two shear tractions, respectively, while $t_{n}^{0}$, $t_{s}^{0}$, and $t_{t}^{0}$ denote the interlaminar normal strength and two shear strengths. The Benzeggagh–Kenane criterion (BK) [25] is employed to predict the initiation of interlaminar damage in composite materials.

### 3.2. Finite Element Model

**Figure 2:**
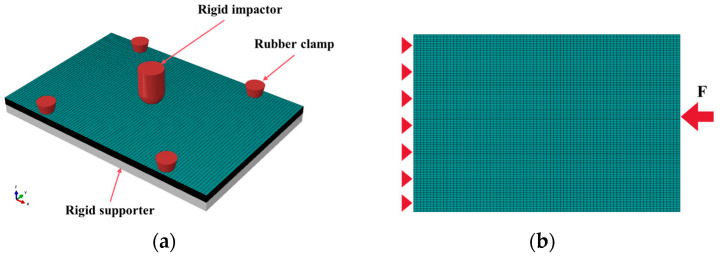
(**a**) Low-velocity impact model; (**b**) CAI model.

A finite element model of bio-inspired helical composite structures under multiple impact loads was established using ABAQUS 2022. Since the low-velocity impact on composites constitutes a dynamic problem characterized by strong nonlinearity and complex contact behavior, the ABAQUS explicit solver was employed for the solution. The intralaminar damage model described in Section 3.1 was implemented via a VUMAT subroutine to determine the initiation and evolution of fiber and matrix damage within plies. A mesh sensitivity analysis was performed to establish the appropriate element size, with the final mesh refined to 1.875 mm in the longitudinal direction and 1 mm in the transverse direction of the laminate, ensuring result precision while maintaining reasonable computational costs. The laminate was discretized using 8-node linear hexahedral reduced integration elements (C3D8Rd). To simulate interlaminar damage evolution, 0.01 mm-thick 8-node cohesive elements (COH3D8s) were inserted between plies. During low-velocity impact simulation, the impactor was modeled as rigid using R3D4 elements considering its negligible strain, while the contact interaction between the impactor and laminate surface was accurately modeled through the general contact algorithm, with a friction coefficient of 0.2. To enhance computational efficiency while maintaining solution accuracy, mass scaling was employed with a target time increment of less than $5\times 10^{-8}$. The finite element analysis procedure comprised three main steps: first, a single-impact ABAQUS model was developed to obtain the initial impact damage characteristics of the bio-inspired helical composite laminate. Second, velocity boundary conditions were adjusted to ensure the impactor’s velocity met the prescribed requirements for subsequent impacts, with the velocity boundary subsequently deactivated—this step could be repeated directly for multiple impacts. Finally, after completing low-velocity impact simulations, the impact boundary conditions were replaced with clamped CAI boundary conditions to further investigate the residual compressive performance of the impacted bio-inspired helical composite laminate. The mesh model for CAI maintained consistency with the impact simulation, ensuring continuous damage transfer from impact to CAI analysis. The bottom of the laminate was assigned a simply supported condition, while the top surface was coupled to a reference point for the CAI load application. The compressive force *F* was then applied to this reference point. The established finite element model is shown in Figure 2.

## 4. Experimental Results and Discussion

### 4.1. Repeated Impact Response

**Figure 3:**
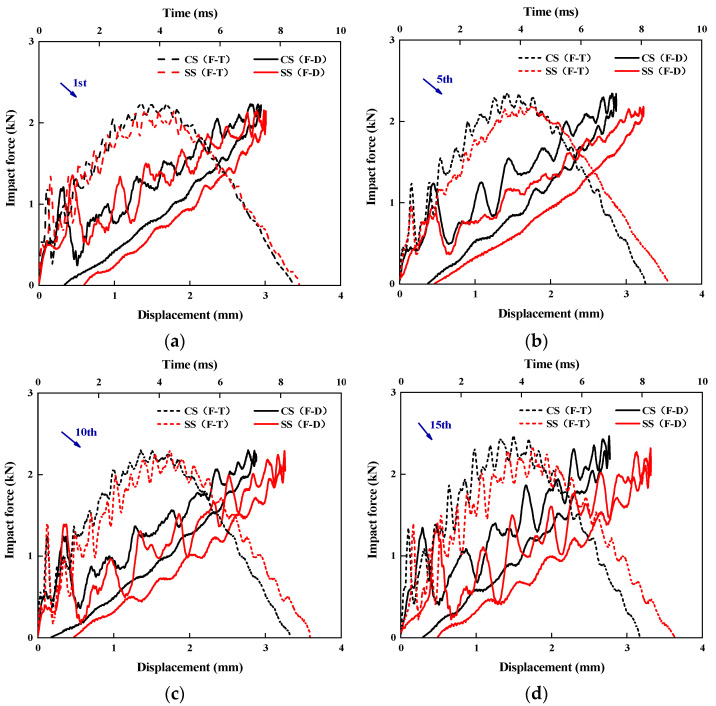
The impact–time/displacement curves of CS and SS laminates for the (**a**) 1st, (**b**) 5th, (**c**) 10th, and (**d**) 15th impact times under 5 J of impact energy.

Figure 3 displays the impact force–time and impact force–displacement curves of cross-spiral laminates (CSs) and symmetric-spiral laminates (SSs) under a 5 J impact energy, subjected to 1, 5, 10, and 15 impact times. The curves of both types of laminates exhibit significant oscillatory characteristics, which are primarily attributed to two factors: the instability of the impactor component connections and the inherent frequency effects caused by the bending vibration of the impacted specimens. A comparative analysis reveals that as the number of impacts increases, the bending stiffness of the SS laminates shows a notable decline, while the impact duration significantly lengthens. Particularly after 15 impacts, the peak force of the SS laminates decreases more substantially compared to that of the CS laminates. This phenomenon fully demonstrates that under impact loading, the unique layup design of the CS laminates can more effectively distribute stress, delaying the initiation and progression of local damage, thereby exhibiting superior resistance to structural deformation.

**Figure 4:**
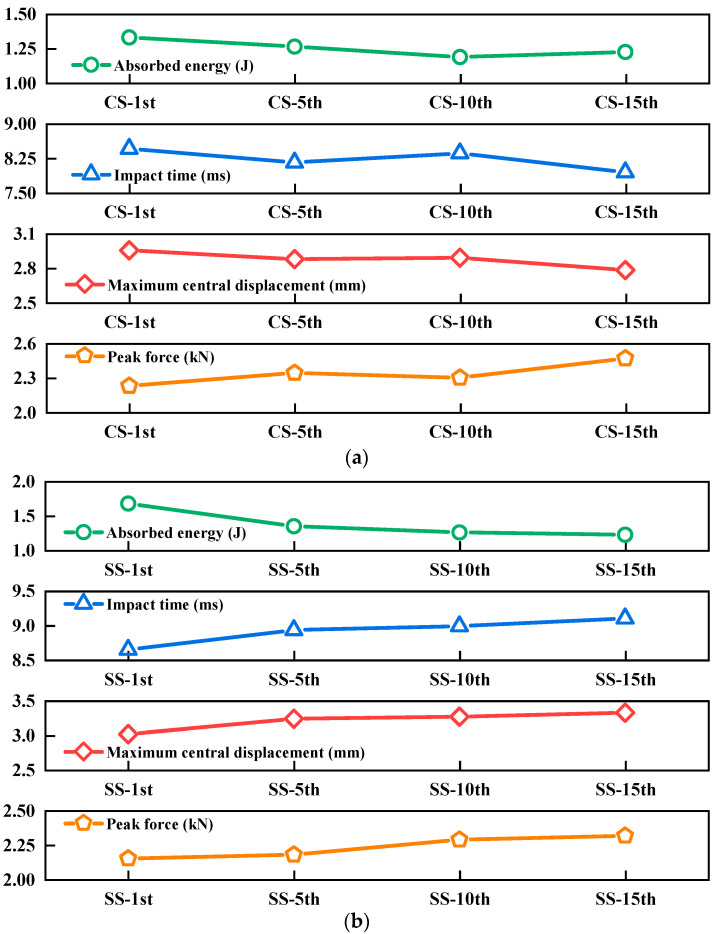
Impact resistance performance of (**a**) CS and (**b**) SS laminates for the 1st, 5th, 10th, and 15th impact times under 5 J of impact energy.

To facilitate the comparison of the mechanical responses of CS and SS laminates under 5 J of impact energy at different impact counts, Figure 4 was plotted. Standard deviation analysis was first performed for all parameters in Figure 4. As shown in Table 3, the relatively small errors observed in all parallel experiments demonstrate high experimental precision, confirming the reliability of our results. Additionally, comparative *t*-test analyses were conducted between CS and SS configurations under 15 J of impact energy at different impact counts (1, 3, 5, and 15), examining the peak force, maximum central displacement, impact time, and energy absorption characteristics (see Table 4 for complete results). The dynamic responses of the two configurations exhibited distinct variations with increasing impact cycles. A significant difference in absorbed energy was observed between the configurations at the first impact (*p* < 0.05), but this disparity gradually diminished with subsequent impacts. Notably, the most pronounced differences emerged at 15 impacts, where both the maximum central displacement and impact duration showed statistically significant variations (*p* < 0.05). However, the peak load consistently demonstrated no statistically significant difference between the configurations throughout the testing (*p* > 0.05).

The results of Figure 4 indicate that the energy absorption capacity and peak force of both types of laminates follow similar trends with increasing impact counts: energy absorption shows a decreasing trend, while the peak force gradually increases. This phenomenon can be attributed to the following mechanisms: as the number of impacts accumulates, both types of laminates develop varying degrees of internal damage, leading to a decline in the energy-absorption capacity. Meanwhile, matrix cracking induced by the initial impact enhances the stiffness of the fiber-reinforced layers, resulting in a progressive increase in peak force during subsequent impacts. Notably, the impact duration of CS laminates fluctuates only within a small range with increasing impact counts, remaining relatively stable overall. In contrast, the impact duration of SS laminates is significantly prolonged as the number of impacts rises. Since the duration of impact contact force is positively correlated with the damage severity, this difference suggests that damage evolution is more pronounced in SS laminates. Furthermore, by analyzing the trend of maximum central displacement, it was found that the displacement of CS laminates remains largely stable, whereas that of SS laminates exhibits a clear upward trend with increasing impact counts. This result further confirms that the bending stiffness of SS laminates undergoes significant degradation after multiple impacts, substantially compromising their deformation resistance.

### 4.2. Non-Destructive Testing

**Figure 5:**
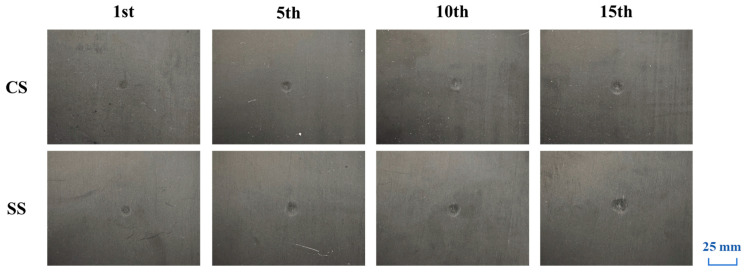
Visual inspection of CS and SS laminates under 5 J repeated impacts (1st, 5th, 10th, 15th).

Figure 5 presents a comparative analysis of the visual damage morphology of CS and SS laminates after multiple impacts under 5 J of impact energy. Experimental observations reveal that both types of laminates exhibit significant local deformation characteristics after a single low-velocity impact, manifested as a distinct permanent circular indentation on the surface of the impact zone. This phenomenon confirms that the material undergoes considerable plastic deformation during impact and effectively absorbs the impact energy. As the number of impacts increases, the damage morphology of both laminates evolves as follows: the area of the indentation zone gradually expands, and the depth of the indentation continues to increase. Notably, under the same number of impacts, the SS laminates demonstrate more severe damage characteristics compared to the CS laminates, with a larger damaged area and more pronounced deformation. This comparative result visually reflects that the SS laminates exhibit a more prominent damage accumulation effect under repeated impact loading.

**Figure 6:**
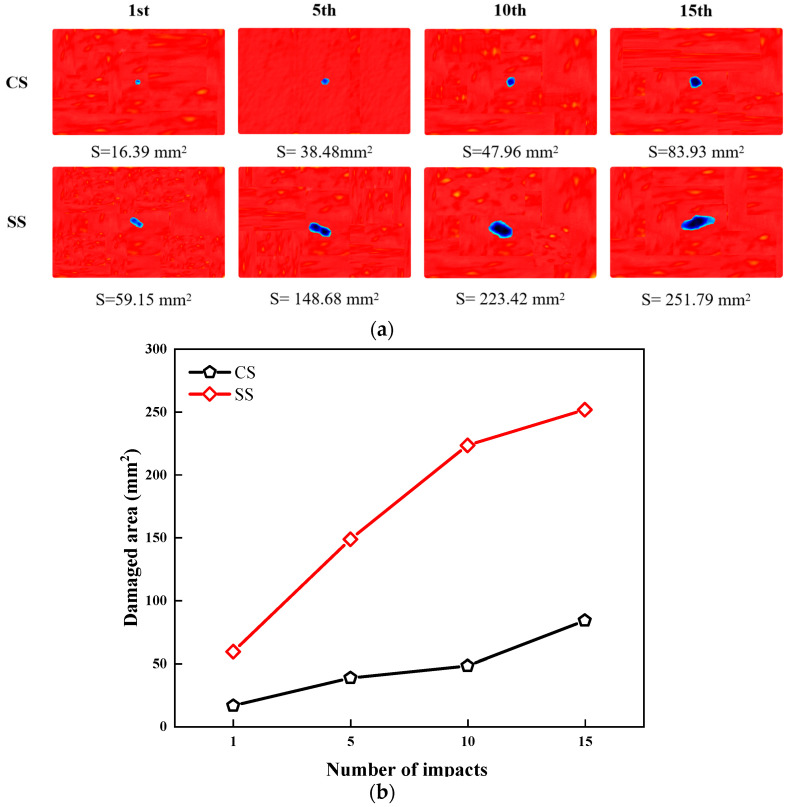
(**a**) Ultrasonic C-scan results; (**b**) damaged area of CS and SS laminates under 5 J repeated impacts (1st, 5th, 10th, 15th).

The damage-propagation behavior after low-velocity impact was characterized using ultrasonic C-scan technology. Figure 6a displays the projected damage contours of the two bionic laminates under different impact counts. The results reveal that, due to differences in the layup configurations, the damage contour of the CS laminate exhibits an approximately circular distribution, while the SS laminate shows a distinct elliptical damage pattern. Figure 6b provides a quantitative comparison of the damage area evolution for both types of laminates. As the number of impacts increases, the damage area of both plates demonstrates a monotonic upward trend, primarily attributed to repeated impacts exacerbating fiber-matrix interface debonding and promoting continuous delamination propagation. Notably, the damage area of the SS laminate is consistently and significantly larger than that of the CS laminate, with this discrepancy further amplifying as the impact count rises. Specifically, after 15 impact times, the damage area of the SS laminate reaches 251.79 mm^2^, approximately three times that of the CS laminate (83.93 mm^2^). This comparative result conclusively demonstrates that the layup configuration design of the CS laminate offers superior impact resistance and damage-suppression capabilities.

**Figure 7:**
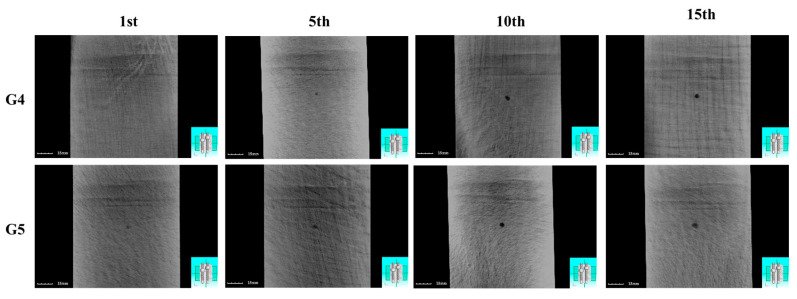
X-ray CT scans of CS and SS laminates under 5 J repeated impacts (1st, 5th, 10th, 15th).

To further analyze the internal damage characteristics of the laminated plates, X-ray CT scanning was performed on both configurations after multiple impacts. Figure 7 presents the CT cross-sectional images at a depth of 0.1 mm from the impact surface under varying impact counts. The analysis reveals that neither laminate exhibits significant damage features at this depth layer after the initial impact. However, as the number of impacts increases, the damage severity intensifies markedly, ultimately forming distinct circular hole-shaped damage in both cases.

### 4.3. CAI Behavior

**Figure 8:**
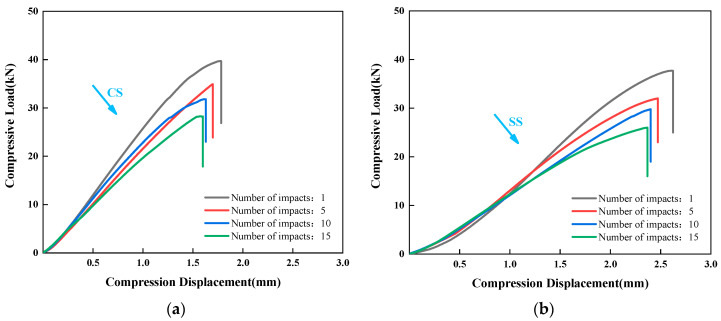
Comparison of compressive load-displacement curves for (**a**) CS and (**b**) SS laminates.

To evaluate the damage tolerance of the two bio-inspired laminate configurations after multiple low-velocity impacts, post-impact compression tests were conducted. As shown in the comparative analysis in Figure 8, with increasing impact counts, minor cracks, delamination, and fiber fractures progressively accumulate in the laminates. This damage accumulation leads to a reduction in both the overall stiffness and load-bearing capacity of the composite, resulting in a gradual decrease in the maximum compressive load. When the impact count reaches five or more, the declining trend of the maximum compressive load gradually slows down as the damage approaches saturation. Particularly, the damage in the surface and near-surface layers has accumulated to a certain extent, and the additional damage caused by subsequent impacts becomes relatively minor. Consequently, the maximum compressive load stabilizes and exhibits a plateau trend.

**Figure 9:**
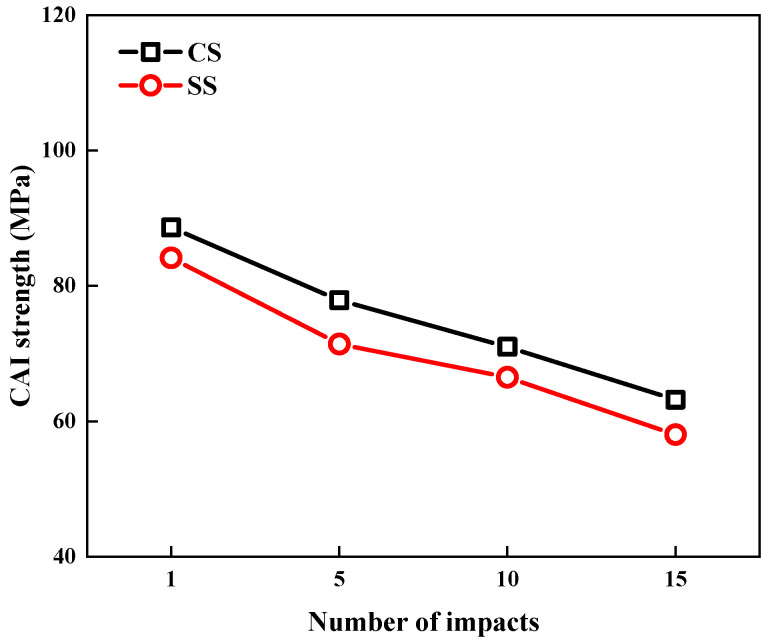
Comparison of CAI strength of CS and SS laminates under 5 J of repeated impacts (1st, 5th, 10th, 15th).

Figure 9 provides a direct comparison of the compressive residual strength (CAI) between CS and SS laminates after different impact counts (1, 5, 10, and 15 impacts) under 5 J of impact energy. Experimental data reveal that while both types of laminates exhibit a significant linear negative correlation between the CAI strength and impact counts, they demonstrate distinct performance characteristics: the CS laminates maintain higher CAI strength values across all impact counts, demonstrating superior damage-tolerance properties. This performance difference conclusively demonstrates the advantages of the CS layup configuration in terms of impact damage resistance and structural integrity preservation. The enhanced performance primarily stems from the unique cross-spiral structure of CS laminates, which more effectively disperses impact energy and suppresses damage propagation.

## 5. Numerical Results and Discussion

### 5.1. Validation of Numerical Model

**Figure 10:**
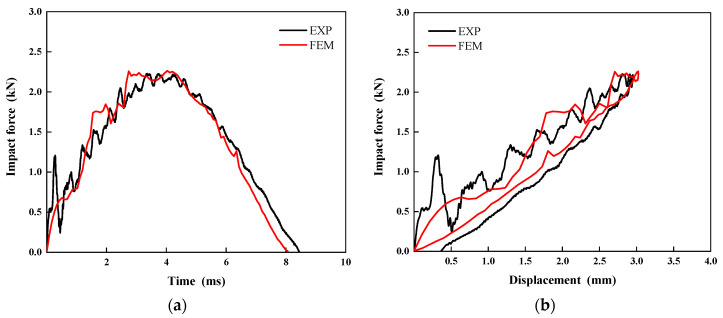
Numerical predictions of the (**a**) impact force–time responses and (**b**) impact force–displacement responses compared with the experimental data for CS configuration.

**Figure 11:**
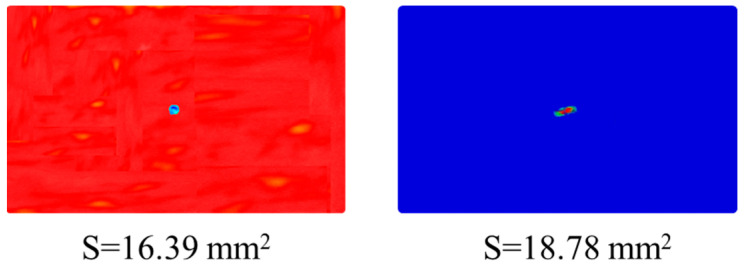
Comparison of experimental and numerical results for impact damage morphology of the CS laminate at 5 J of impact energy.

**Figure 12:**
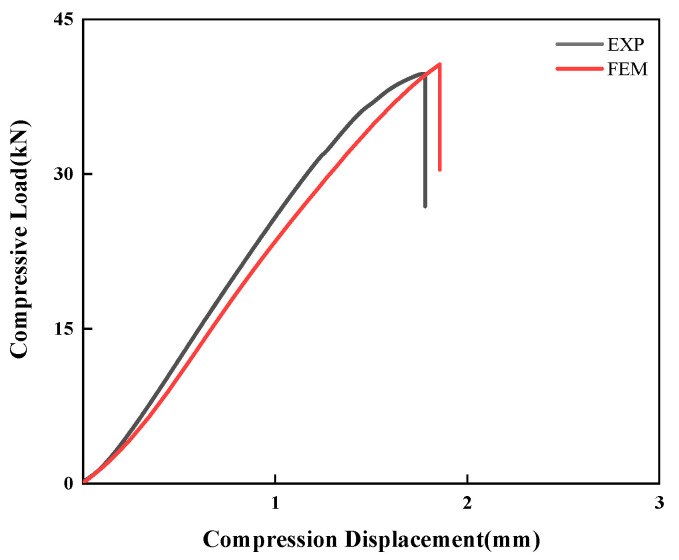
Numerical predictions of the compressive load–compressive displacement responses with the experimental results for the CS configuration.

To validate the accuracy of the established numerical model, this study conducted a comparative analysis between experimental and simulation results for CS laminates under a single 5 J impact. As shown in Figure 10, the numerically simulated impact force–time and impact force–displacement curves demonstrate excellent agreement with the experimental measurements. This confirms the finite element model’s capability to accurately characterize the dynamic response of laminates under impact loading. Furthermore, Figure 11 presents a comparison between C-scan measurements and finite element predictions of damage morphology in CS laminates. A quantitative analysis reveals that the numerical model precisely captures both the extent and morphological characteristics of the damage zone. The predicted damage area of 86.7 mm^2^ shows only 14.6% relative error compared to the experimental results, meeting the engineering analysis accuracy requirements. In terms of CAI performance prediction, the comparative results in Figure 12 demonstrate that the numerical model not only accurately reproduces the load-displacement response curve of the laminate during compression, but also shows a mere 2.3% deviation between the predicted CAI strength (90.70 MPa) and the experimentally measured value (88.62 MPa). This series of comparative results fully validates the reliability of the established numerical model in predicting the impact response, damage evolution, and residual strength.

### 5.2. Optimal Design of Laminates

Based on the validated finite element model, this study designed three additional bio-inspired laminated structures, in addition to the original CS and SS configurations. As shown in Table 5, the bio-inspired configurations include a double-spiral structure (DS), linear–cross structure (LC), and linear–spiral structure (LS). Using numerical simulation methods, a comparative analysis was conducted to examine the differences in impact response characteristics and damage tolerance between these three new configurations and the previously studied CS laminate, which exhibited superior performance.

**Figure 13:**
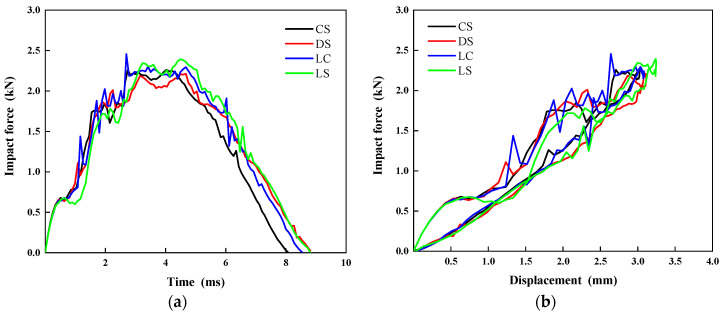
Comparison of the (**a**) impact force–time history and (**b**) impact force–displacement curves for the four different configurations at 5 J of impact energy.

As shown in Figure 13, a comparative analysis of the impact force–time and impact force–displacement curves of four bionic configuration laminates under 5 J of impact energy reveals that the mechanical responses of different configuration laminates exhibit similar trends. Among them, the LS laminate demonstrates the best impact resistance, achieving the highest peak force, while the DS laminate shows the weakest impact resistance relatively with the lowest peak load. In terms of the impact duration, the CS laminate displays the fastest impact response, with a duration of only 8.1 ms; in contrast, the DS and LS laminates have the longest impact durations, both reaching approximately 8.8 ms. Additionally, the stiffness analysis results indicate that the CS and LC laminates possess comparable bending stiffness, which is significantly superior to that of the DS and LS laminates.

**Figure 14:**
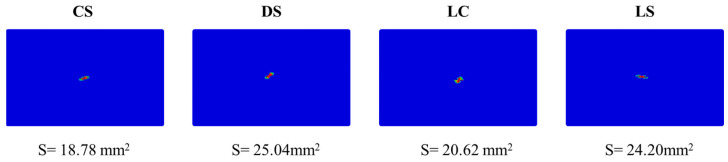
Comparison of the impact damage morphology predicted by numerical model for the four different configurations at 5 J of impact energy.

Figure 14 compares the damage morphology and damage area of the four configuration laminates under 5 J of impact energy. The results show that although the damage morphology of all configurations exhibits an elliptical-like pattern, their forming directions differ significantly due to variations in ply angles. Notably, under single-impact conditions, the CS laminate displays the smallest damage area (18.78 mm^2^), whereas the DS laminate has a damage area of 25.04 mm^2^, representing an increase of approximately 33.3% compared to the CS laminate.

**Figure 15:**
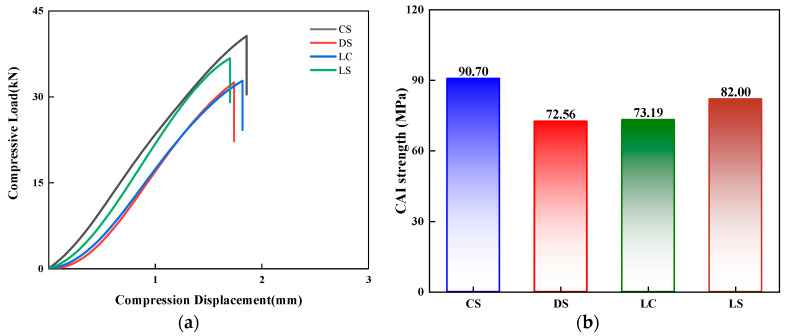
Comparison of the (**a**) compressive load–compressive displacement responses and (**b**) CAI strength predicted by the numerical model for the four different laminate configurations at 5 J of impact energy.

Figure 15 presents a comparative analysis of the damage tolerance performance of the four configuration laminates. As shown in Figure 15a, the load–displacement curves of all four laminates exhibit similar overall trends: the load increases continuously with compressive displacement until final fracture failure occurs at approximately 2 mm of displacement. A comparison of CAI strength (Figure 15b) reveals that the CS-configuration laminate demonstrates the most superior damage-tolerance performance, achieving a CAI strength of 90.70 MPa, which is significantly higher than the other three configurations. Notably, the DS and LC configuration laminates exhibit comparable CAI strength, both maintaining around 73 MPa. The comprehensive findings indicate that the CS-configuration laminate exhibits outstanding advantages in both impact resistance and damage tolerance, showcasing the best overall mechanical performance.

## 6. Conclusions

This study adopts a combined approach of experimental research and numerical simulation to investigate the multiple-impact resistance and damage tolerance characteristics of glass fiber-reinforced bionic composite laminates. The main conclusions are as follows:

Under repeated impact loading, the CS-configuration laminate demonstrates significant performance advantages. Compared with the SS configuration, the CS configuration exhibits markedly superior impact resistance and damage tolerance, with this advantage becoming increasingly pronounced as the number of impacts grows.

The finite element model established based on continuum damage mechanics theory effectively predicts the impact resistance and damage tolerance of CS laminates. Through virtual drop-weight impact tests and CAI tests, the key mechanical response data, such as impact force-time/displacement curves and CAI strength curves were successfully obtained, with simulation results showing good agreement with experimental data.

The study also comparatively evaluated the performance differences among the four bionic laminate configurations. While all configurations exhibited comparable impact resistance, the CS configuration demonstrated notable advantages in damage tolerance, further validating its potential as an excellent structural material.

## Figures and Tables

**Table 1:** Laminate configuration.

Laminate Code	Number of Layers	Stacking Sequence	Stacking Angle (°)
CS (Cross-Spiral)	32	[G_32_]	[0/90/6/84/12/78/18/…/78/12/84/6/90/0]
SS (Symmetric-Spiral)			[0/6/12/18/…/84/90]_s_

**Table 2:** Material properties for glass fiber lamina.

Materials	Glass Fiber Lamina
Density (kg/m^3^)	ρ=2000
Modulus (GPa)	$E_{11}=62,\;E_{22}=E_{33}=7.5$ $G_{12}=G_{13}=3.6,\;G_{23}=3.2$
Poisson’s ratio	$\upsilon_{12}=\upsilon_{13}=0.32,\;\upsilon_{23}=0.39$
Strength (MPa)	$X_{T}=750,\;X_{C}=420$ $Y_{T}=58,\;Y_{C}=152$ $S_{12}=S_{13}=65,\;S_{23}=52$
^a^ Intralaminar fracture energy (N/mm)	$G_{ft}=18,\;G_{fc}=2,\;G_{mt}=0.4,\;G_{mc}=1.8$
^a^ Interface modulus (GPa)	E=5
^a^ Interface strength (MPa)	N=S=30
^a^ Interface fracture energy (N/mm)	$G_{n}^{C}=0.6,\;G_{s}^{C}=2.1$

^a^ Based on the Reference [20], predicted value in this study.

**Table 3:** Standard deviations of impact resistance for CS and SS laminates at 5 J of impact energy during 1st, 5th, 10th, and 15th impacts.

Laminate	Peak Force/kN	Maximum Central Displacement/mm	Impact Time/ms	Absorbed Energy/J
CS-1st	0.164	0.140	0.443	0.096
CS-5th	0.313	0.285	0.350	0.074
CS-10th	0.116	0.150	0.189	0.089
CS-15th	0.133	0.163	0.372	0.065
SS-1st	0.108	0.170	0.131	0.094
SS-5th	0.104	0.160	0.142	0.068
SS-10th	0.167	0.386	0.279	0.084
SS-15th	0.151	0.124	0.171	0.101

**Table 4:** *t*-test analysis of laminates with CS and SS configurations.

	*p*	Peak Force	Maximum Central Displacement	Impact Time	Absorbed Energy
Number of Impacts					
1		0.467	0.103	0.421	0.001
5		0.359	0.570	0.006	0.110
10		0.927	0.058	0.003	0.257
15		0.178	0.001	0.001	0.948

**Table 5:** Design bionic laminate configuration.

Laminate Code	Number of Layers	Stacking Sequence	Stacking Angle (°)
DS (Double-Spiral)	32	[G_32_]	[0/6/12/18/…/84/90] + [0/6/12/18/…/84/90]
LC (Linear–Cross)			[0/6/12/18/…/84/90] + [0/90/6/84/12/78/18/72/24/ 66/30/60/36/54/42/48]
LS (Linear–Spiral)			[0/6/12/18/…/84/90/96… 180/186]

## Data Availability

The raw/processed data required to reproduce these findings cannot be shared at this time as the data also forms part of an ongoing study.
